# Crosslinked HA in Topical Application: The Biological Benefits of RHA Serum on Aging Signs

**DOI:** 10.1111/jocd.71065

**Published:** 2026-07-22

**Authors:** Romain Brusini, Laure Peslerbe, Camille Vantou, Pauline Meriaux, Lesley Rose Clark‐Loeser, Jimmy Faivre

**Affiliations:** ^1^ Research and Development Department Teoxane SA Geneva Switzerland; ^2^ Teoxane Aesthetic Skincare Geneva Switzerland; ^3^ Precision Skin and Body Institute Davie Florida USA

**Keywords:** anti‐aging, collagen production, cosmetics, crosslinked hyaluronic acid, serum

## Abstract

**Background:**

RHA Serum is a topical formulation with proven benefits on skin hydration and biomechanical properties. This study aims to evaluate its role in remodeling the extracellular matrix by stimulating the production of endogenous hyaluronic acid and collagens, thereby supporting its clinical efficacy in reducing visible signs of aging.

**Methods:**

Anti‐aging effects were assessed using ex vivo human skin explants and a 56‐day, split‐face, randomized clinical study. Abdominoplasty‐derived human skin explants were treated with RHA Serum or controls and analyzed histologically and immunohistochemically for HA and collagen types I and IV expression. In parallel, 22 women aged 40–65 years applied RHA Serum or placebo daily. Skin thickness and dermal density were measured by high‐frequency ultrasound, while skin quality parameters were quantified using Visia digital imaging.

**Results:**

Ex vivo analysis revealed significantly increased expression of HA and collagens I and IV in RHA Serum‐treated samples. In vivo, RHA Serum significantly improved dermal density and epidermal thickness compared to a placebo cream. Visia analysis further confirmed visible reductions in facial wrinkles and skin roughness, aligning with biological improvements observed ex vivo.

**Conclusion:**

RHA Serum supports skin structure by promoting key extracellular matrix components, including collagens and endogenous HA. These biological effects translate into measurable clinical improvements. The findings support RHA Serum as a potent, evidence‐based anti‐aging topical treatment.

## 1 Introduction

Skin aging is a multifactorial process driven by both intrinsic and extrinsic mechanisms [1]. Intrinsic aging reflects the natural, genetically programmed decline in physiological function over time, characterized by increased reactive oxygen species production, reduced cellular repair capacity, and cellular senescence [2, 3, 4, 5]. Extrinsic aging is largely influenced by environmental and lifestyle factors, notably ultraviolet (UV) radiation and pollution, which collectively accelerate the structural deterioration of the skin [1]. Together, these factors lead to a progressive loss of structural integrity and physiological function. Histologically, aging skin exhibits a thinner epidermis and dermal‐epidermal junction (DEJ) flattening, which compromises epidermal‐dermal cohesion [6], accompanied by degradation of the extracellular matrix (ECM) [3] including a marked decline in collagen content and glycosaminoglycans, particularly hyaluronic acid (HA) [7]. Clinically, these changes manifest for example as skin dryness, reduced firmness and elasticity, or the appearance of fine lines and wrinkles [1].

As global life expectancy rises, maintaining youthful skin has become a major societal, scientific, and economic priority. Anti‐aging interventions now target all layers of the skin and span a wide spectrum, from topical agents to minimally invasive procedures such as botulinum toxins, soft‐tissue fillers, and biostimulators, all with demonstrated clinical efficacy [3, 8]. Among these, HA remains a cornerstone ingredient in both medical and cosmetic dermatology due to its exceptional hydrating properties [7, 9]. Free HA continues to be widely used in topical formulations [10, 11], despite its limited skin penetration depending on its molecular weight [12, 13], and short half‐life. A notable advancement in this area was the development of RHA (Resilient Hyaluronic Acid) Serum, a topical formulation that incorporates crosslinked HA similar to the one found in soft‐tissue injectables [14, 15]. This formulation is designed to offer prolonged hydration and improve skin biomechanical properties, such as firmness and elasticity. Comparative studies have demonstrated that RHA Serum outperforms conventional high‐ and low‐molecular‐weight free HA in reducing transepidermal water loss, enhancing epidermal hydration, and improving corneocyte structure. These effects were observed as early as the first application and sustained over 28 days, alongside significant improvements in skin quality [14, 15].

This article explores the biological and clinical performance of RHA Serum through a two‐pronged approach: first, by evaluating its impact on key ECM elements, including HA and collagens, using an ex vivo human skin model; and second, through skin quality measurements from a blinded, split‐face clinical study. Collectively, these findings provide a comprehensive assessment of RHA Serum's potential to restore youthful skin characteristics through a non‐invasive, daily topical routine.

## 2 Materials and Methods

### 2.1 Preparation of HA‐Based Formulations and Control Treatments

Crosslinked Resilient Hyaluronic Acid (RHA, Teoxane Laboratories, Switzerland) was synthesized under sterile conditions using high molecular weight sodium hyaluronate (HTL Biotechnology, France) and 1,4‐butanediol diglycidyl ether as a crosslinking agent, yielding a final 25 mg/mL hydrogel after dilution in phosphate buffer supplemented with free sodium hyaluronate.

RHA Serum (Teoxane Laboratories, Switzerland) is a commercial dermocosmetics product containing 3% (w/w) crosslinked RHA and additional hydrating active ingredients. RHA was tested as the main ingredient of RHA Serum by incorporating it into a 1.5% (w/w) aqueous carboxymethyl cellulose (CMC) gel matrix at a final concentration of 3% (w/w). A CMC solution at the same concentration (15 mg/g) was prepared as a “vehicle” control. Lastly, a “Base cream” composed of Aqua, *Helianthus Annuus* Seed Oil, Mineral oil, Emulsifier, Preservatives, and perfume was used as comparison during the clinical trial.

### 2.2 Evaluation of the Anti‐Aging Activity of RHA Serum on Human Skin Explants

#### 2.2.1 Ex Vivo Study Design

Human skin samples obtained from surgical wastes (with patient consent) were used to evaluate the ex vivo anti‐aging activity of RHA Serum, in compliance with the Declaration of Helsinki and French Public Health Code (Article L.1243–4). Experiments were conducted in collaboration with BIO‐EC Laboratory (Eurofins) following internal protocols.

Twelve human skin explants (average diameter 11 ± 1 mm) comprising the dermal and epidermal layers (fat tissue removed) were prepared from an abdominoplasty of a 47‐year‐old Caucasian woman with a Fitzpatrick phototype II. Explants were maintained in BIO‐EC's Explants culture Medium (BEM) at 37°C in a humid, 5%‐CO_2_ atmosphere.

#### 2.2.2 Ex Vivo Treatment Protocol

At baseline (D0), three explants were processed as plasty controls. For the other conditions, explants were cultured and processed after 10 days of treatment. Skin explants were distributed into four experimental groups of three explants each: untreated control explants (No Treatment), explants treated with 1.5% CMC gel (vehicle), explants treated with RHA at 3% in 1.5% CMC gel (RHA 3%), and explants treated with RHA Serum. Tested products were applied topically at the rate of 2 μL per 1 cm^2^ (≈2 mg/cm^2^) on D0, D1, D2, D3, D6, and D8. The control explants did not receive any treatment. The BEM culture medium was half renewed for every condition on D2, D3, D6, and D8.

#### 2.2.3 Histological Processing of Skin Explants

On D0 and D10, three explants per batch were collected and cut into two parts. Half were fixed in 4% buffered formalin solution for 24 h, dehydrated, paraffin‐embedded, and sectioned at 5 μm using a Leica RM 2125 microtome (Leica Biosystems, Germany). The other half were frozen at −80°C in OCT medium and sectioned at 7 μm using a Leica CM 3050 cryostat. All sections were stained for biomarkers analysis, then mounted on Superfrost plus silanized histological slides (ThermoFisher Scientific, USA), and examined microscopically using Leica DMLB or Olympus BX43/BX63 (Evident Scientific, Japan) microscopes. Images were acquired with Olympus DP74 cameras and processed with CellSens software (Evident Scientific, Japan).

#### 2.2.4 Image Analyses and Semi‐Quantification of Biomarkers of Interest

Formalin‐fixed sections were immunostained for HA using biotinylated HABP (AMSBIO, UK; 1:400 in PBS, 1 h, RT) and a biotin/streptavidin amplification system, visualized with VIP substrate (Vector Laboratories, USA). Frozen sections were immunostained with polyclonal antibodies for collagen I (Abcam, UK; 1:1600) and collagen IV (Southern Biotech, USA; 1:200), diluted in PBS containing 0.3% BSA and 0.05% Tween 20 for 1 h at RT.

Alexa Fluor 488‐conjugated secondary antibodies (Lifetechnologies, USA) were used for detection, and nuclei were counterstained with propidium iodide. Image analyses were performed using CellSens software, by delineating regions of interest (ROIs) and quantifying by thresholding the percentage of stained area within each ROI. Results are expressed as mean ± SE from five ROIs per condition.

### 2.3 Clinical Assessment of the Efficacy of RHA Serum on Skin Quality

#### 2.3.1 Clinical Study Design and Protocol

A clinical study was conducted to evaluate the efficacy of RHA Serum in face care, in compliance with international ethical standards for medical research involving human subjects (Declaration of Helsinki, 1964, and subsequent amendments). Twenty‐two healthy female volunteers aged 40–65 years, presenting slight to moderate wrinkles, were recruited under the supervision of a board‐certified dermatologist after providing written informed consent.

The study was carried out according to a randomized, single‐blind, split‐face design. Hemi‐face treatment allocation was determined by a computer‐generated randomization sequence using Wei's urn algorithm (PASS 11 software, NCSS LLC, USA), ensuring balanced allocation across subjects. One randomly assigned hemi‐face received RHA Serum and the contralateral side received a placebo cream (termed Base cream) composed exclusively of formulation excipients: emollient base ( *Helianthus Annuus* Seed Oil, Isononyl Isononanoate), standard emulsifiers (Polyglyceryl‐3 Methylglucose Distearate, Glyceryl Stearate, Cetearyl Alcohol), chelating agent (Disodium EDTA), preservatives (Chlorphenesin, O‐Cymen‐5‐OL), and fragrance, with no biologically active ingredients. Subjects were blinded to the product applied to each hemi‐face throughout the study duration. At baseline, skin thickness and dermal collagen content were quantified by means of high‐frequency ultrasound system (DUB SkinScanner, Taberna Pro Medicum GmbH, Germany), and standardized digital images were captured using the Visia‐CR system (Canfield Scientific, USA). Patients applied the assigned formulations twice daily from D0 through D56. Ultrasound measurements and Visia‐CR imaging were subsequently repeated at D28 and D56 to assess longitudinal changes in skin biophysical parameters.

#### 2.3.2 Clinical Evaluation of Skin Thickness and Dermis Density

All procedures were standardized in accordance with ISO 9001 quality management guidelines. Instrumental evaluations were performed under controlled environmental conditions (18°C–26°C; 50% ± 10% Relative Humidity), following a 15–20 min acclimatization period. Measurements were acquired using the DUB SkinScanner, non‐invasive 50 MHz high‐resolution ultrasound device. Cross‐sectional scans (5 mm width × 5 mm depth) were captured at the zygomatic region of the face, yielding a 50 μm axial resolution. Epidermal–dermal thickness and dermal backscatter intensity (as a proxy for density) were quantified, and for each time point, the three best‐quality scans per patient were analyzed.

#### 2.3.3 Visualization of the Clinical Benefits of the Treatments

Facial images were acquired using the Visia‐CR system, using multi‐modal illumination (standard, cross‐polarized, UV) to optimize visualization of surface and subsurface skin features. Wrinkle depth was quantified from transversal line profiles by measuring distances between the normal skin surface and the wrinkle valley. Skin roughness was assessed within a rectangular ROI by analyzing color histograms and calculating the standard deviation as an indicator of surface irregularity.

In the absence of standardized cosmetic reference values, efficacy was defined as improvement in clinical endpoints in more than 50% of participants.

#### 2.3.4 Statistical Analysis

Statistical analyses data were performed using the Kruskal–Wallis test with a Dunn's test post hoc pairwise comparisons (ex vivo data) or the paired *t*‐test (Clinical data comparison of RHA Serum versus base cream) in OriginPro 2023 (OriginLab, USA). The differences were considered statistically significant at *p* < 0.1 (*), *p* < 0.05 (**), *p* < 0.01 (***), and *p* < 0.001 (****) in all studies. Values were expressed as Mean ± Standard Error when applicable.

## 3 Results

### 3.1 Topical Application of RHA Serum Promotes the Production of HA in Papillary Dermis

**FIGURE 1:**
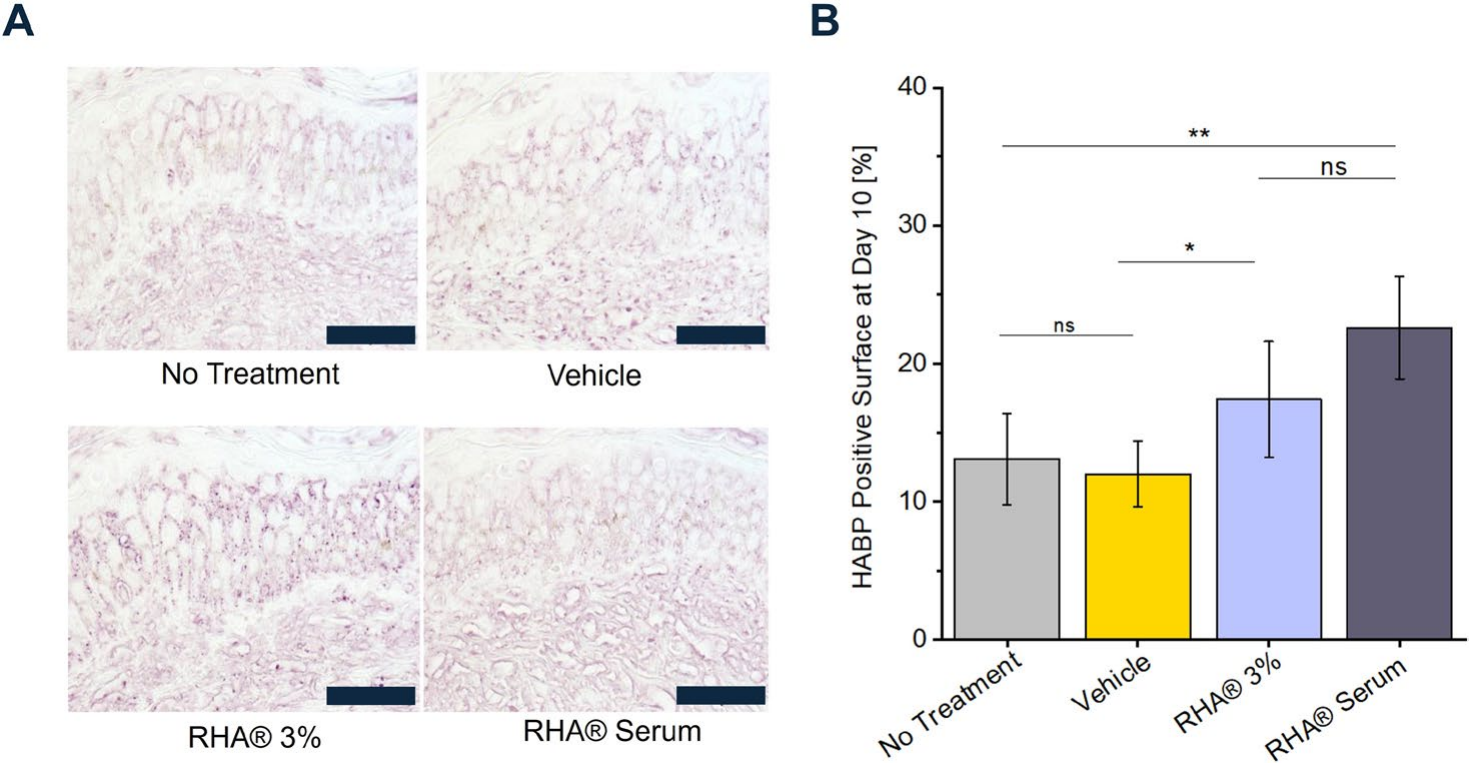
HABP expression measured after 10 days of topical treatment on human skin explants (*N* = 3). (A) Representative images of HABP immunostaining on human skin sections centered around the dermal‐epidermal junction (DEJ). Scale bars = 50 μm. (B) Semi‐quantitative analysis of HABP‐positive surface area in papillary dermis. Results are expressed as mean ± SE. Statistical significance was assessed using the Kruskal–Wallis test with Dunn’s post hoc pairwise comparisons. Significance levels are indicated as *p* < 0.1 (*), *p* < 0.05 (**).

The effect of topical application of RHA 3% and RHA Serum on HA production within the dermis was evaluated using human skin explants over a 10‐day period using vehicle‐treated and untreated samples as controls. HA content was visualized using hyaluronic acid binding protein (HABP) immunostaining (Figure 1A). It revealed a strong HABP signal localized predominantly within the viable epidermis and the upper dermal layers (papillary dermis), with a concentrated signal around the DEJ. Topical treatments of RHA 3% and RHA Serum markedly influenced the distribution and intensity of HABP expression. Compared with controls, the epidermis exhibited more intense HABP distribution throughout the suprabasal keratinocytes, particularly with RHA 3% treatment. In the dermis, moderate to dense HABP immunoreactivity was observed surrounding fibroblast cells, suggesting enhanced hyaluronan organization and turnover following treatment, more specifically with RHA Serum.

Quantitative analysis was performed as the percentage of HABP‐positive area within the defined ROI (Figure 1B). The vehicle exhibited a mean positive surface area of 12.0% ± 2.4%, which was not significantly different from the untreated control (13.1% ± 3.3%). In contrast, the RHA 3% condition, containing crosslinked HA dispersed in the cellulose‐based vehicle for the sake of topical delivery, significantly enhanced HA presence (17.4% ± 4.2%) compared to the vehicle alone. The treatment with the RHA Serum also resulted in a statistical increase in dermal HA levels, with HABP‐positive areas reaching 22.6% ± 3.7%. HA levels induced by RHA 3% were statistically comparable to those achieved with the RHA Serum, underpinning the efficacy of RHA 3% as the key ingredient of the serum in boosting HA deposition in the upper dermal layer.

### 3.2 Increased Collagen Expression in Human Skin Explants Following Topical Treatment With RHA Serum

**FIGURE 2:**
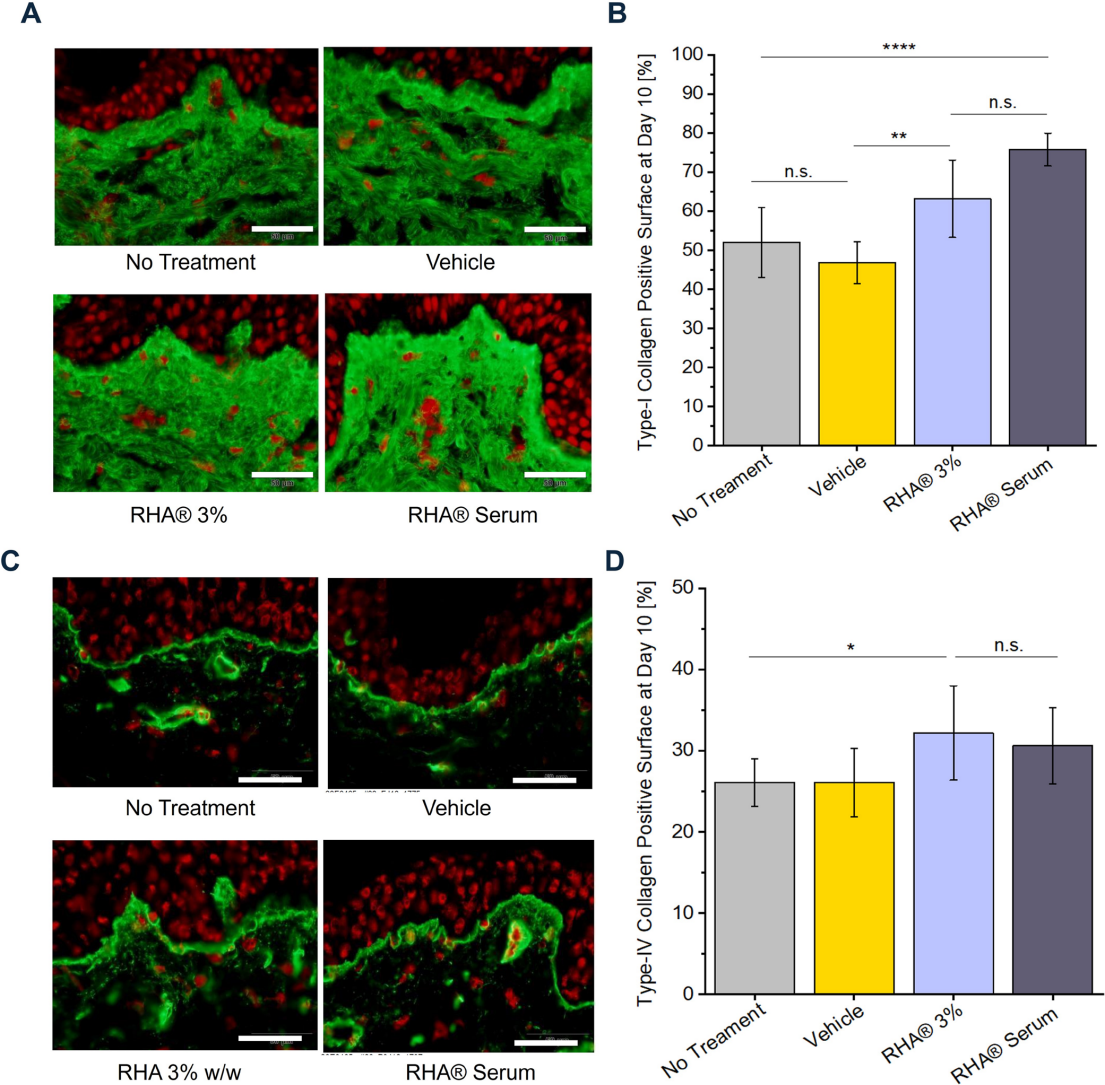
Collagen expression in human skin explants following 10‐day topical treatment (*N* = 3). (A) Representative immunofluorescence images showing Type I collagen localization in the papillary dermis. Scale bars = 50 μm. (B) Semi‐quantitative analysis of Type I collagen‐positive surface area. (C) Representative immunofluorescence images showing Type IV collagen localization at the DEJ. Scale bars = 50 μm. (D) Semi‐quantitative analysis of Type IV collagen‐positive surface area. Results are presented as mean ± SE. Statistical significance was assessed using the Kruskal–Wallis test with Dunn’s post hoc pairwise comparisons. Significance levels are indicated as *p* < 0.1 (*), *p* < 0.05 (**), *p* < 0.01 (***), *p* < 0.001 (****).

Figure 2 illustrates the expression of collagen in the upper layers of human skin explants after 10 days of topical application of RHA 3% and RHA Serum. Immunostaining was performed to specifically detect type I and type IV collagens, two key structural proteins involved in dermal integrity and skin regeneration. As shown in Figure 2A, type I collagen was distributed throughout the dermal layer, with the highest density of signal observed directly subjacent to the DEJ. In both control groups, fluorescence intensity appeared visually weaker and less homogeneously distributed compared to the RHA 3% and RHA Serum‐treated groups. Semi‐quantitative analysis (Figure 2B) confirmed that the cellulose‐based vehicle did not significantly differ from the untreated control (46.8% ± 5.3% vs. 52.0% ± 9.0%, respectively). In contrast, RHA 3% treatment significantly increased type I collagen expression (63.2% ± 9.9%) as well as RHA Serum, which showed even higher type I collagen levels (75.8% ± 4.2%), although the difference from RHA 3% alone was not statistically significant.

Type IV collagen, primarily localized at the DEJ, showed a similar trend. As depicted in Figure 2C, both RHA 3% and RHA Serum treatments resulted in visibly stronger fluorescence intensity and a thicker type IV collagen layer compared to controls. Although these differences did not reach statistical significance (Figure 2D), a clear upward trend was observed compared to both control groups.

### 3.3 Clinical Evaluation of RHA Serum Using High‐Frequency Ultrasound Imaging and 3D‐Digitial Imaging

**FIGURE 3:**
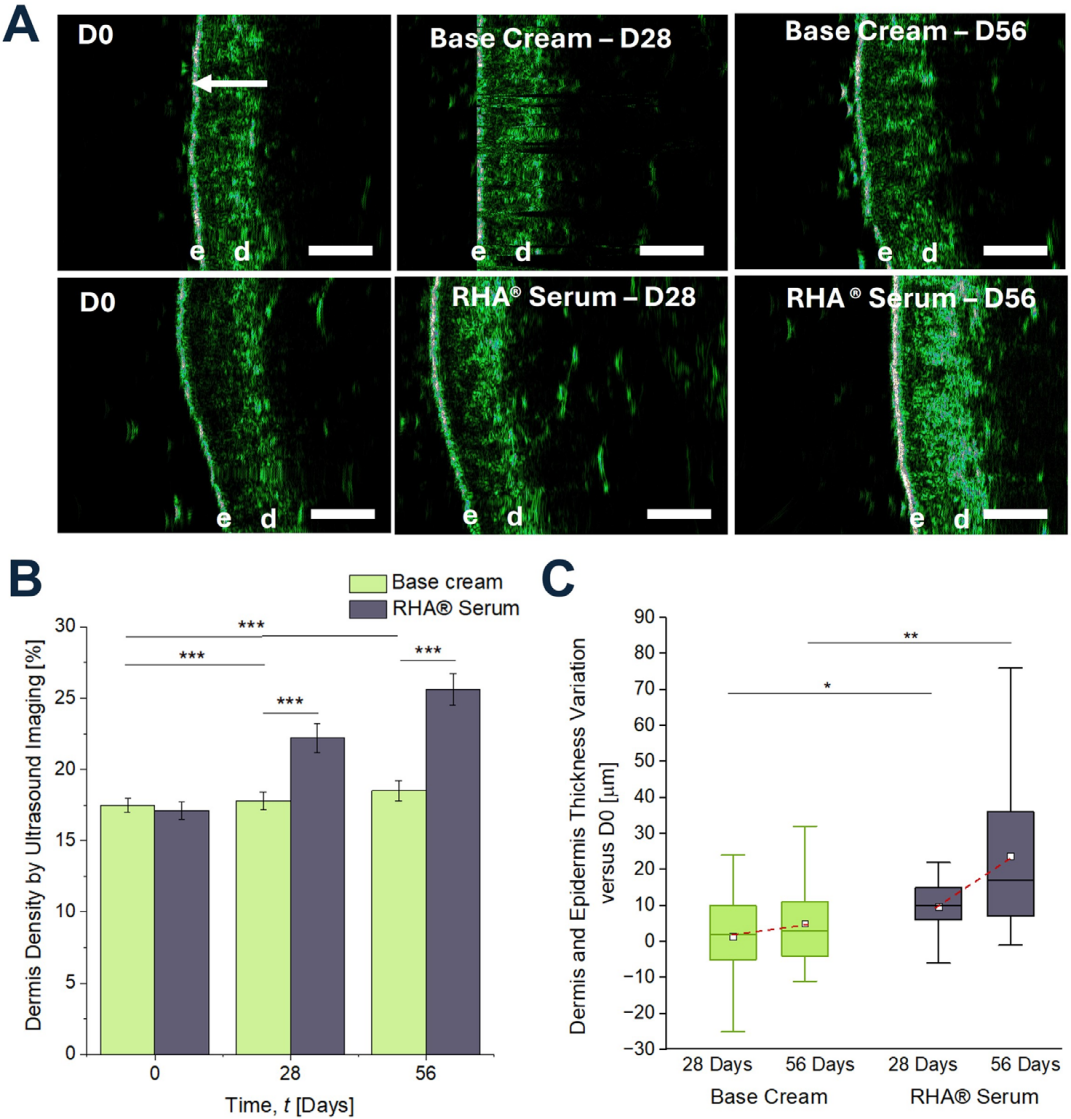
Ultrasound‐based evaluation of dermal and epidermal changes over 2 months following daily application of RHA Serum versus a base cream (*n* = 22). (A) Representative ultrasound images of the cheekbone area on both sides of the face (RHA Serum versus base cream) at D0, D28, and D56 in one patient. The white arrow delimitates the segment of the skin ultrasound images used for the measurement of dermis density and dermis + epidermis thickness. Scale bar = 1000 μm. (B) Quantitative analysis of dermis density over time across all patients. (C) Variation in dermis + epidermis thickness relative to baseline. White squares represent mean values; box plots show interquartile ranges (25th–75th percentiles) around the median. Dashed red lines illustrate the mean evolution over time. Results are expressed as mean ± SE. Statistical significance was assessed using a paired *t*‐test. Significance levels are indicated as *p* < 0.1 (*), *p* < 0.05 (**), *p* < 0.01 (***).

RHA Serum was evaluated in a clinical study involving 22 patients using a split‐face design. High‐frequency ultrasound imaging was performed on the cheekbone area at baseline D0, D28, and D56 to assess dermis density and combined epidermis‐dermis thickness (Figure 3). At baseline, the epidermis appeared as a thin hyperechoic band, while the dermis was moderately echogenic and thicker in structure (Figure 3A). Over time, the echogenicity remained relatively stable on the base cream‐treated side, whereas the RHA Serum‐treated side showed increased echogenicity, indicative of a denser tissue structure. Quantitative analysis of dermis density is presented in Figure 3B. At D0, both sides exhibited comparable dermal density (RHA Serum: 17.1% ± 0.6%; base cream: 17.5% ± 0.5%). After 28 days of treatment, the RHA Serum demonstrated a statistically significant increase in dermis density compared to base cream (22.2% ± 1.0% vs. 17.8% ± 0.6%, respectively). This difference further intensified by D56, reaching 25.6% ± 1.1% for RHA serum versus 18.5% ± 0.7% for the base cream. Regarding skin thickness (Figure 3C), baseline measurements were similar between both sides (RHA serum: 1419.3 ± 29.2 μm; base cream: 1428.1 ± 36.2 μm). The base cream induced minimal thickening over time (Day 28: +1.5 μm; n.s., Day 56: +5.0 μm, n.s.). In contrast, the RHA Serum led to a statistically significant increase at both timepoints (Day 28: +9.6 μm, *p*‐value < 0.05; Day 56: +23.9 μm, *p*‐value < 0.01).

**FIGURE 4:**
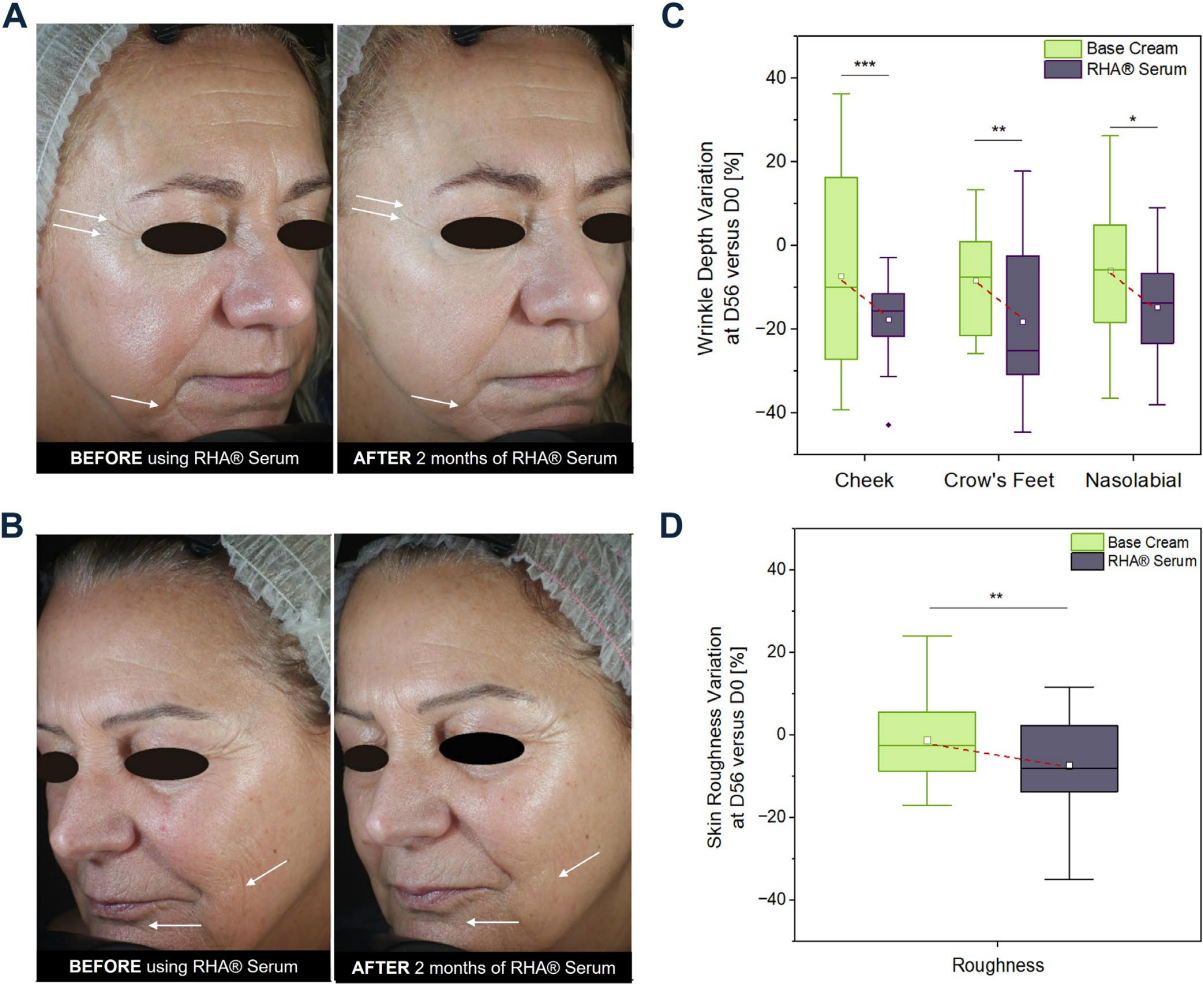
Standardized, 3D‐digital imaging of patient (A) and patient (B) before and after twice‐daily application of RHA Serum for 56 days (parallel polarized images). Arrows indicate areas of notable improvement in wrinkle severity. (C) Percentage variation in wrinkle depth at D56 relative to baseline, for the cheek, crow's feet, and nasolabial folds, as assessed by 3D‐digital imaging. (D) Percentage variation in skin roughness at D56 relative to baseline measured via 3D‐digital imaging. White squares represent mean values; box plots display interquartile ranges (25th–75th percentiles) around the median. Dashed red lines illustrate the mean evolution over time.

Figure 4A,B present two case reports illustrating the clinical effects of RHA Serum over a two‐month treatment period. Patient A exhibited a marked reduction in wrinkle length and depth in the periorbital and perioral regions. Digital imaging revealed improvements in skin smoothness, a reduction in redness, and enhanced radiance, suggesting a multifaceted benefit of RHA Serum on overall skin appearance. Patient B showed a visible decrease in the wrinkles surface area in the perioral region, along with a significant improvement in skin texture and luminosity. Further quantitative assessments were performed using Visia‐CR imaging technology. Figure 4C displays wrinkle depth in three distinct regions: crow's feet, nasolabial folds, and cheeks. Statistically significant improvements were observed in all regions. In the cheek area, RHA Serum reduced wrinkle depth by 17.1%, compared to 7.5% with the base cream (*p* < 0.01). In the crow's feet region, RHA Serum achieved an 18.1% reduction versus 8.3% with the base cream (*p* < 0.05). In the nasolabial area, RHA Serum showed a 15.2% reduction compared to 4.3% with the base cream (*p* < 0.1). Finally, in a comparative evaluation of skin roughness, RHA Serum demonstrated a statistically significant improvement, with a reduction of 7.4% from baseline, compared to the base cream which exhibited a 1.1% decrease (*p* < 0.05).

## 4 Discussion

RHA Serum is a topical skincare formulation developed to improve skin quality and hydration as part of a daily routine. It contains 3% (w/w) of crosslinked HA (RHA), adapted from soft‐tissue filler technology, and designed to support skin barrier function. Topical application of HA‐based products has previously been shown to enhance skin hydration [16, 17], particularly when using crosslinked HA rather than free high‐ or low‐molecular‐weight HA. Notably, its ability to form a long‐lasting film on the skin surface reduces transepidermal water loss and promotes moisture retention [11, 14, 15]. Beyond its reported hydrating action, the present study demonstrated that RHA Serum induces significant biological and clinical improvements in aging skin. Particularly, application of RHA Serum led to a measurable upregulation of types I and IV collagens, as well as endogenous HA expression in superficial skin layers, although measured on one skin donor. Nevertheless, these molecular changes corresponded to statistically significant skin structure enhancements observed clinically after 1 month, where high‐frequency ultrasound imaging revealed increased epidermal‐dermal thickness and dermis density.

These findings mirror prior reports demonstrating HA‐based serums can stimulate endogenous HA synthesis, notably via upregulation of hyaluronan synthase‐2 [18] along with increased collagen production, contributing to visible esthetic benefits [9, 18]. Besides, the stimulation of fibroblast [9, 18] activity and improved ECM organization observed right below the epidermis in the treated explants indicate that RHA Serum may modulate dermal homeostasis beyond passive hydration, potentially through mechano‐transduction or signaling effects at the dermal‐epidermal junction. These observations align with previous clinical studies that have further supported the efficacy of topical HA serums in reducing fine lines and improving skin texture [16, 17], as well as described their capacity to support fibroblast viability, collagen remodeling, and tissue cohesion [15].

Similarly, the current patient‐centered data demonstrated that twice‐daily use of RHA Serum for 2 months resulted in clinically visible improvements in wrinkle depth, skin smoothness, and overall quality, corroborating the ex vivo results. These findings are consistent with previously published data on skin biomechanical properties [15], documenting a 16% increase in skin firmness and a 25% enhancement in elasticity after 2 weeks of twice‐daily application of RHA Serum compared to no treatment.

Furthermore, the base cream employed as control does not induce comparable enhancements, thereby validating the beneficial effects are specifically attributable to the active ingredients of RHA Serum. Particularly, the RHA 3% contained in the formulation induced a marked increase in ECM components expression, validating the main bioactivity of RHA in topical form. RHA Serum further amplified these effects, although not significantly, suggesting a synergistic combination with complementary hydrating and bioactive ingredients within the serum formulation. It may especially be attributed to the presence of Disodium Acetyl Glucosamine Phosphate, documented to optimize cutaneous water retention [19, 20, 21, 22] and Alteromonas Ferment extract, a novel ingredient recognized for its modulatory effects on ECM remodeling [23]. The present results thus extend current understanding of HA‐based topical efficacy by showing that a crosslinked HA network, traditionally designated for injectables, can elicit meaningful biological and structural effects when applied topically.

## 5 Conclusion

This study demonstrates that RHA Serum significantly improves key biological and clinical markers of skin aging. By supporting the expression of endogenous hyaluronic acid and collagens I and IV, it promotes epidermal and dermal remodeling and reinforces skin structure. Clinically, these effects translate into measurable improvements in skin density, thickness, and reduction of visible signs of aging such as fine lines and roughness.

These results highlight not only the effectiveness of RHA Serum, but also the broader relevance of advanced skincare in modern anti‐aging strategies. As a scientifically validated, non‐invasive topical solution, RHA Serum supports skin quality enhancement through targeted stimulation of the extracellular matrix. Its ability to improve both biological and visible signs of aging makes it a valuable complement to more intensive esthetic treatments, offering a holistic and synergistic approach to skin rejuvenation.

## Author Contributions

R.B. participated in the conceptualization, methodology, validation, and data analysis, writing of the original draft, and visualization. L.P. participated in the conceptualization, methodology, validation and data analysis, writing of the original draft, and coordinated the clinical trial. C.V. participated in the conceptualization, methodology, data analysis, and writing of the draft. P.M. participated in the conceptualization, methodology, and analysis of preclinical data. L.R.C.‐L. participated in the formal analysis of clinical data and the writing of the original draft. J.F. participated in the conceptualization, validation, visualization, formal data analysis, writing of the original draft, allocating resources, and supervising the overall project.

## Funding

The authors have nothing to report.

## Consent

An informed consent for publication of photographs has been obtained from the patients.

## Conflicts of Interest

R.B., L.P., C.V., P.M., and J.F. are employees of Teoxane SA, Switzerland. RHA Serum is manufactured by Teoxane SA. The remaining authors declare no conflicts of interest.

## Data Availability

The data that support the findings of this study are available on request from the corresponding author. The data are not publicly available due to privacy or ethical restrictions.
